# Early Symptoms in Children with Inflammatory Bowel Disease: Implications for Subsequent Bone Mineral Deficiency

**DOI:** 10.3390/children11101223

**Published:** 2024-10-09

**Authors:** Mariusz Olczyk, Agnieszka Frankowska, Marcin Tkaczyk, Anna Socha-Banasiak, Elżbieta Czkwianianc

**Affiliations:** 1 Department of Pediatrics, Immunology and Nephrology, Polish Mother’s Memorial Hospital Research Institute, Medical University of Lodz, 90-419 Lodz, Poland; agnieszka.frankowska@stud.umed.lodz.pl (A.F.);; 2 Department of Gastroenterology, Allergology and Pediatrics, Polish Mother’s Memorial Hospital Research Institute, 93-338 Lodz, Poland

**Keywords:** IBD, children, Crohn’s disease, ulcerative colitis, manifestations, metabolic bone disorders, bone mineral density

## Abstract

Background: Inflammatory bowel disease (IBD) is associated with multiple factors that influence bone metabolism. This study aimed to compare the clinical manifestations and diagnostic parameters of patients with Crohn’s disease (CD) and ulcerative colitis (UC) at the time of diagnosis, as well as to assess their relationship with subsequent bone disorders. Methods: Blood tests (including calcium–phosphate metabolism) and fecal tests (including calprotectin) were performed in eighty children recently diagnosed with IBD. Additionally, the bone densitometry results were evaluated in 25 of them. Results: Diarrhea (*p* = 0.02) and bloody stools (*p* < 0.001) were more frequent in patients with UC, whereas fever was more common in patients with CD (*p* = 0.003). Laboratory tests revealed anemia in 62.5% (50/80) and thrombocytosis in 36.3% (29/80). Higher calprotectin levels in the feces were found in girls at the time of diagnosis (*p* = 0.02). Osteopenia was detected in almost half of the examined patients (12/25), and 20% (5/25) met the criteria for osteoporosis. Low calcium levels at diagnosis were correlated with subsequent bone disorders (*p* = 0.005). Insufficient levels of vitamin D were detected in 77.8% (56/80). Conclusions: Early disease detection and the appropriate monitoring of children with IBD may decrease the risk of serious consequences, including osteoporosis.

## 1. Introduction

Inflammatory bowel disease (IBD) is increasingly prevalent among children globally, especially in regions with historically lower incidence rates [1,2]. Despite a well-established clinical understanding of these chronic gastrointestinal disorders, the surge in pediatric cases poses challenges due to the multifaceted and nonspecific symptoms that often arise, leading to diagnostic uncertainties.

**Scheme 1:**
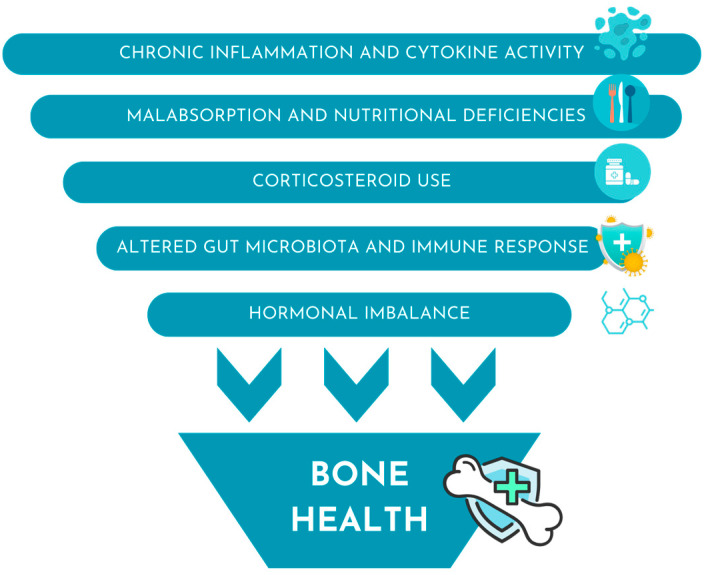
Underlying mechanisms of bone metabolic disorders in pediatric inflammatory bowel diseases.

While Crohn’s disease (CD) and ulcerative colitis (UC) share similarities in clinical presentation, progression, and prognosis between pediatric and adult populations, children with IBD face an elevated risk of extraintestinal symptoms. The intricate balance of the immune system in young individuals can result in various consequences, such as growth disorders, anemia, and deficiencies in essential vitamins and minerals like A, B12, D, E, selenium, zinc, copper, and calcium [3,4,5,6]. Research has shown that in pediatric IBD patients, there is a significant reduction in dendritic cells, which is associated with an imbalance between regulatory T cells and Th17 cells. The abovementioned correlation is crucial for maintaining immune tolerance and controlling inflammation [7]. Moreover, the impact of systemic pro-inflammatory cytokines, steroid therapy, and nutritional and vitamin deficiencies on bone metabolism is a recognized concern [8,9], yet the intersection of bone metabolic disorders and children with IBD remains insufficiently explored. Potential pathways contributing to bone metabolic disorders are presented in Scheme 1.

Given that adolescence is a critical phase for skeletal development, overlooking symptoms in youth may culminate in long-term repercussions, potentially leading to conditions like osteoporosis in adulthood [10]. This study seeks to compare the clinical manifestations and diagnostic parameters of CD and UC in children at the time of diagnosis. Additionally, it aims to explore the correlation between these initial characteristics and the subsequent development of bone disorders. Understanding these relationships is crucial for early intervention strategies that can mitigate the long-term impact of IBD on skeletal health in children.

## 2. Materials and Methods

This retrospective study included children hospitalized in the Clinic of Gastroenterology, Allergology, and Pediatrics at the Polish Mother’s Memorial Hospital Research Institute in Lodz between 1 January 2014 and 3 January 2023. The inclusion criteria were as follows: diagnosis of Crohn’s disease or ulcerative colitis based on clinical presentation, endoscopy, and histopathological examination of intestinal mucosal biopsy.

Out of 131 evaluated patients, 51 patients were excluded from the analysis because of an insufficient number of markers in the compared laboratory tests (*n* = 22), concurrent chronic diseases (*n* = 5) such as anorexia (which may disrupt the credibility of the obtained results), or ongoing observations for non-specific intestinal inflammation (*n* = 24). Patients with congenital bone mineral disorders were not taken into consideration in the study.

According to obtained anthropometric measurements (body weight, height, BMI), the results were plotted on percentile charts. Weight loss was assessed based on routine check-ups performed by a primary care physician. Inflammatory markers (C-reactive protein—CRP and erythrocyte sedimentation rate—ESR), blood morphology parameters (white blood cell count, hemoglobin concentration, platelet count, mean corpuscular volume of red cells), iron concentration in the blood, calcium–phosphate metabolism (including total calcium concentration, phosphate levels, and regulatory substances such as parathyroid hormone, vitamin D, and alkaline phosphatase), magnesium and creatinine concentrations in the blood, thyroid hormones (TSH, FT4), and calprotectin concentration in the stool were analyzed. Vitamin D deficiency is defined as a 25(OH)D concentration below 20 ng/mL (50 nmol/L), while “vitamin D insufficiency” is defined as a concentration of 25(OH)D in the range of 21–29 ng/mL (525–727 nmol/L) [8]. Bone density was also assessed by dual-energy X-ray absorptiometry (DXA) using the Z-Score Total Body and Z-Score L1–L4. Gastrointestinal symptoms and extraintestinal manifestations were evaluated. Under consideration were the parameters noted when the diagnosis was made. All patients included in the study were treated according to current guidelines for managing IBD, and all received corticosteroid therapy.

All of the obtained results were statistically analyzed using the Statistica program (data analysis software system), version 13 [(TIBCO Software Inc. (2017), Mumbai, Maharashtra]. Correlations were assessed using the Spearman Test, and statistical differences between the groups were evaluated using non-parametric tests such as Mann–Whitney U. Statistical significance was set at *p* < 0.05. Approval from the Bioethics Commission was obtained for the implementation of the study (RNN/09/23/KE from 10 January 2023).

## 3. Results

### 3.1. The Basic Characteristics of the Population

The final study group consisted of 80 IBD patients aged 4 to 18 years (mean age 12.4, SD ± 3.55, median 12), including 35 girls (mean age 12.5, SD ± 3.86, median 13) and 45 boys (mean age 12.3, SD ± 3.28, median 12). Children diagnosed with UC accounted for 66.3% (53/80) of the patients, while CD accounted for 33.7% (27/80). Based on the PUCAI scores, 35.8% (*n* = 19) had mild disease, 41.5% (*n* = 22) had moderate disease, and 22.7% (*n* = 12) had severe disease. According to the PCDAI scores 20% (*n* = 5) had mild disease, 52% (*n* = 13) had moderate disease, and 28% (*n* = 7) had severe disease.

### 3.2. Clinical Presentation of IBD

An analysis of the patients’ medical documentation revealed that, at the time of diagnosis, the most common symptom was abdominal pain, which was observed in 65/80 (81.3%) children with IBD. The second most common was diarrhea at 60/80 (75%), followed by bloody stools in 52 out of 80 (65%), weight loss in 26/80 (32.5%), constipation in 12/80 (15%), and fever in 11 out of 80 (13.8%). Some rare symptoms such as general weakness (*n* = 5) and skin changes (*n* = 3) were not included in the statistical analysis. Among the children with IBD, fever was more frequent in CD patients (CD 30% vs. UC 6%; *p* = 0.003). In patients diagnosed with UC, bloody stools (CD 15% vs. UC 91%; *p* = 0.001) and diarrhea (CD 59% vs. UC 83%; *p* = 0.02) were significantly more common. Abdominal pain, constipation, and weight loss appeared at a similar frequency in both groups (*p* = 0.95, *p* = 0.49, *p* = 0.83, respectively). The data are presented in Table 1.

### 3.3. Laboratory Parameters

In laboratory tests, anemia was observed in 50/80 patients (62.5%)—the mean hemoglobin concentration was 11.53 g/dL, SD ± 1.64, median 11.8, and thrombocytosis in 29/80 (36.3%) patients (mean platelet count was 414 thousand, SD ± 123, median 395). The mean concentration of calprotectin in stool was 2258.9 mg/kg in children with CD and 3684.0 mg/kg in children with UC, but this difference did not reach statistical significance (*p* = 0.14). However, higher calprotectin concentrations were correlated with higher platelet counts (*p* = 0.02, R = 0.27). Among the cohort of individuals exhibiting heightened acute-phase reactant levels, 43.8% (*n* = 35) displayed elevated C-reactive protein levels, whereas 66.2% (*n* = 53) exhibited increased erythrocyte sedimentation rate levels.

### 3.4. Differences between CD and UC

Statistical analysis showed that patients with CD presented higher levels of acute-phase protein CRP in the blood at the time of diagnosis (*p* < 0.001) and higher levels of ESR (*p* = 0.04). The third statistically significant relationship was related to the red blood cell volume; in the group of patients with CD, the values were lower. A comparison of the analyzed parameters among the patients with IBD is presented in Table 2.

### 3.5. Gender Differences and Age-Related Associations

**Figure 1:**
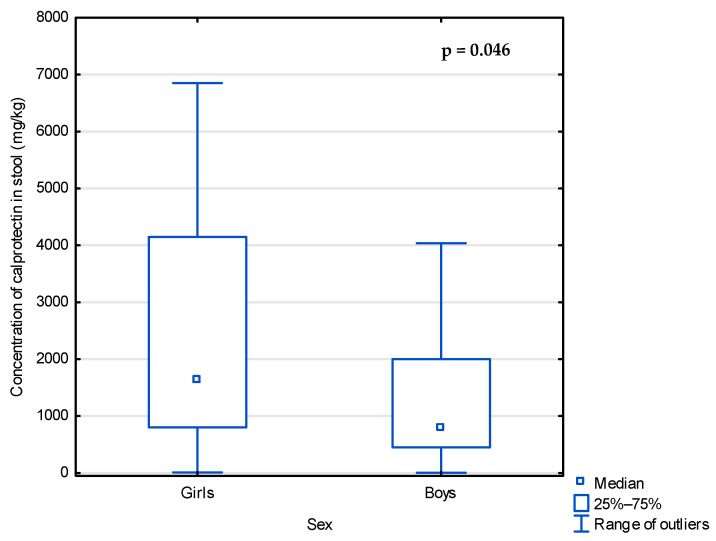
Differences in calprotectin concentration in stool according to child’s gender.

Sex differences within the studied parameters were also observed in the study group. Among girls, a lower concentration of hemoglobin was noted (*p* = 0.01). Contrarily, the level of calprotectin in the stool was higher (*p* = 0.05) (Figure 1).

### 3.6. Bone Mineral Density

Bone density was examined in almost a quarter of the participants within a year of disease diagnosis (22.5%; *n* = 18), with the remaining 8.8% (*n* = 7) being assessed a year or more after diagnosis. The time from diagnosis of the disease to densitometry was usually over half a year (median 208 days). The arithmetic mean Z-Score TB was −1.35 (SD 1.19), and Z-Score SP was −0.65 (SD 1.09). The Z-Score TB ranged from [−3.5 to +1.1], and the Z-Score SP ranged from [−2.7 to +1.8]. In the group of patients who underwent densitometry, nearly half (48%, *n* = 12) had osteopenia. Furthermore, in the case of five children (20% of those who underwent densitometry), the values qualified for a diagnosis of osteoporosis.

**Figure 2:**
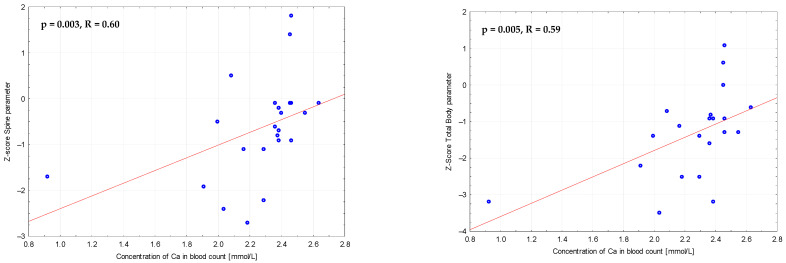
Correlations between concentration of Ca in blood count and Z-Score Total Body and Spine parameters. The blue dots represent the data of our patients, while the red line indicates the trend line.

**Figure 3:**
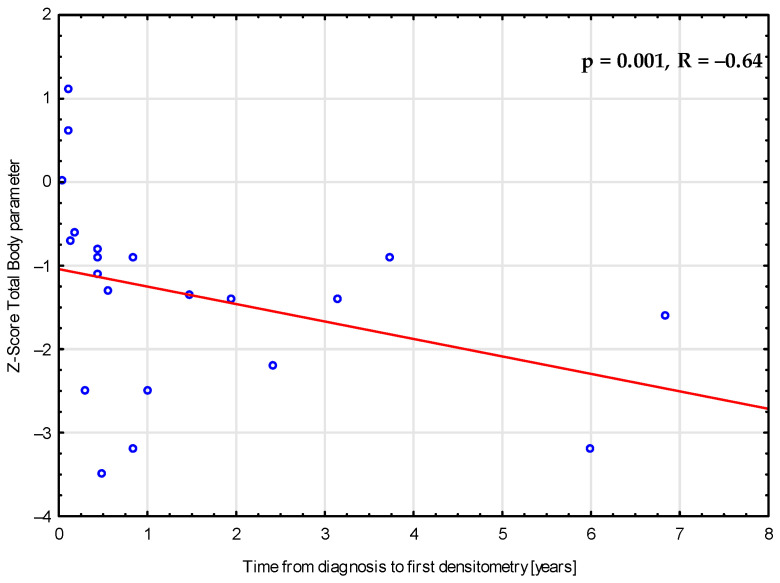
Correlation between time from diagnosis to first densitometry and Z-Score Total Body parameter. The blue dots represent the data of our patients, while the red line indicates the trend line.

A positive correlation was found between low calcium levels at the time of disease diagnosis and low Z-score values in the Total Body (*p* = 0.005, R = 0.59) and Spine (*p* = 0.003, R = 0.60) projections (Figure 2). The later the examination was performed, the statistically lower the Z-score TB values were (Figure 3, *p* = 0.001, R = −0.64). There was no statistically significant difference in bone density between males and females, although in girls, the median Z-Score TB and Spine were lower (boys: median −0.9; −0.3; girls: median −1.4; −0.8). No relationship was found between the vitamin D levels at the time of diagnosis and subsequent bone mineral deficiency.

### 3.7. Vitamin D

**Scheme 2:**
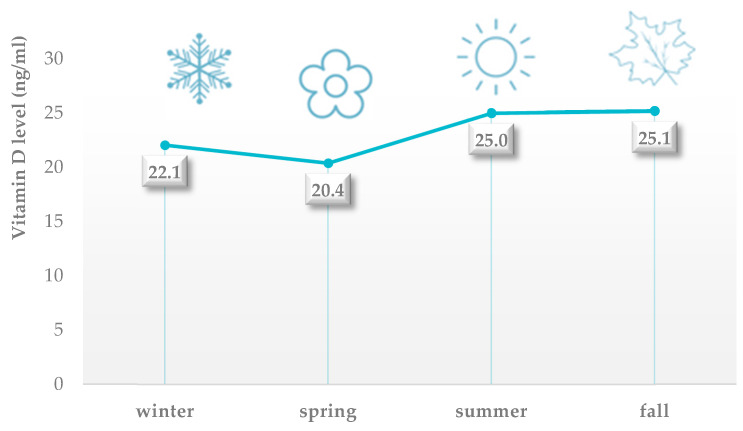
Mean vitamin D levels according to the season of measurement.

In the study group, the mean value of vitamin D concentration was 23.6 ng/mL (SD ± 3.28, median 21.1). A deficiency in 47.2% (*n* = 34) and insufficient concentration of this vitamin in 77.8% (*n* = 56) of patients with IBD were found. A positive correlation was observed between the vitamin D level and serum iron concentration (R = 0.29, *p* = 0.02). However, no correlation between the concentration of vitamin D and inflammatory markers, including calprotectin in the stool, was observed. Differences in the mean concentration of this vitamin were observed depending on the season in which the determination was made. The lowest vitamin D levels appeared among children whose diagnosis was made in spring or winter (20.4 ng/mL and 22.1 ng/mL), with the highest levels observed in summer and autumn (25.0 ng/mL and 25.2 ng/mL). No statistical significance was achieved (Scheme 2, *p* = 0.26).

## 4. Discussion

Differentiating between ulcerative colitis (UC) and Crohn’s disease (CD) poses a challenge to the diagnostic process because of the presence of many common symptoms in both conditions. However, through retrospective analysis and comparisons with results from other publications, we can identify certain differences that may contribute to facilitating and improving diagnosis.

In the work by Moazzami et al. [11], one of the main symptoms observed in patients with both UC and CD was abdominal pain. In our study, we found that this symptom occurred most frequently. Unfortunately, it is a highly non-specific manifestation; therefore, in the diagnosis of inflammatory bowel diseases, it is necessary to consider additional manifestations. Moreover, patients with UC experience bloody stools and diarrhea much more frequently, which aligns with the results described by the aforementioned researchers [11]. These are characteristic symptoms of UC; however, it is worth mentioning that they can also occur in cases of CD. The final diagnosis should be based on an endoscopic examination and histopathological analysis of the collected tissue samples.

In our study, weight loss occurred at a similar frequency in patients with CD and UC (33% and 32%, respectively). Moazzami et al. indicated that weight loss occurs more frequently in patients with CD [11]. Discrepancies in the results may arise from differences in the characteristics of the studied populations or the methods of data analysis, including the assessment of body weight at a certain time after diagnosis. In our study, data were collected during the first hospitalization, regardless of how long the symptoms lasted, which may have influenced the results.

Among the patients with UC, low hemoglobin levels were observed in 29 cases, accounting for 54.7% of the analyzed group. What is more, in 77.8% (*n* = 21) of the children with CD reached diagnostic values indicating anemia. Similar results were observed in another studied group of 165 pediatric patients, which showed that 57% had anemia at diagnosis, dropping to 25% after one year of follow-up [5]. Conversely, in a study by Turkish scientists, anemia was also documented, but only in patients with ulcerative colitis [11]. Discrepancies in the results may arise from differences in the characteristics of the studied populations or the methods of data analysis. It is worth mentioning that among patients with UC, anemia is associated with blood loss, whereas patients with CD suffer from chronic inflammation and absorption disorders that can lead to hematological problems.

Another important aspect worth considering is inflammatory markers. In the group of examined patients, C-reactive protein (CRP) was more frequently elevated in patients with Crohn’s disease (74%), whereas elevated CRP was only observed in 28% of patients with ulcerative colitis. Similar observations were made in the works of other researchers [12,13]. However, CRP should not be the primary diagnostic indicator because of its high sensitivity and potential presence in various other medical conditions such as common infections. Considering this, the next parameter that should be determined to narrow the diagnostic spectrum is the calprotectin level in the stool. In our study, no statistically significant differences were found between calprotectin levels in CD and UC, probably owing to its variable concentration depending on the stage of the disease. However, certain factors predisposing patients to disease development, such as thrombocytosis and female sex, were associated with higher values of this marker at the time of diagnosis. According to the results, this relationship may indicate that, in girls, intestinal inflammation may be more severe, leading to a greater number of complications in the course of IBD. It is worth mentioning that despite being a more expensive test and not providing a definitive diagnosis for either of the diseases studied by us, calprotectin is a more specific parameter for inflammatory bowel diseases and shows a higher correlation with them than CRP [13]. Furthermore, elevated levels can occur up to 3 months before the clinical onset of the disease symptoms [12].

In inflammatory bowel diseases, vitamin D is one of the parameters subject to significant fluctuations depending on various variables, such as genetic factors, season, disease stage, or proper supplementation. Among the children for whom vitamin D levels were determined, deficiency (levels below 20 ng/mL) was found in almost half (47.2%, *n* = 34) and insufficient levels (<30 ng/mL) were identified in 77.8% (*n* = 56) of patients. In our analysis, a positive correlation was observed between vitamin D levels and serum iron concentrations. However, no correlation was found between vitamin D levels and inflammatory markers, including calprotectin in stool. This differs from the results of our previous study [14,15], which may result from taking into account a one-time measurement of vitamin D concentration and not its long-term observation. Seasonal variations in vitamin D levels were also observed, with lower levels in children diagnosed with the disease in spring and winter, and higher levels in summer and autumn. However, statistical significance was not achieved in this study (*p* = 0.26).

Another significant issue worth noting is the direct relationship between inflammatory bowel disease, vitamin D deficiency, and bone metabolism disorders. Patients with IBD have an increased risk of developing metabolic bone disorders, mainly due to chronic inflammation, nutritional deficits, vitamin deficiencies, and steroid therapy [9]. The impaired absorption of fat-soluble vitamins and frequent concomitant lack of appetite during the disease may lead to reduced calcium and mineral intake, which can result in nutritional deficiencies. Steroid therapy alleviates both systemic and local inflammation, which can indirectly lead to bone disorders. Although this therapy is very effective, it significantly affects bone health and development, creating a specific vicious circle [16]. Studies have suggested a possible correlation between serum vitamin D levels and calprotectin levels in the stool of patients with IBD [12]. However, in our study, we did not find statistical significance for this correlation (*p* = 0.68). A significant proportion of the patients (68.8%) did not undergo bone density testing throughout the diagnostic process or, at least, it was not documented in the medical records. Bone health seems to be frequently overlooked, despite numerous studies indicating the occurrence of metabolic bone disorders in patients with inflammatory bowel diseases [15,16,17,18,19]. Even more concerning is the result of our analysis, in which more than half of the patients who underwent bone densitometry had reduced bone density. Comparable results were obtained in a study by Ahn et al. and the Z-scores did not differ between CD and UC groups [20]. A low calcium level recorded at the time of diagnosis should increase physicians’ vigilance regarding this aspect during the entire process of treating the child. Considering that delayed bone densitometry testing in IBD patients is often associated with lower bone density values, the issue of bone metabolism in children with non-specific inflammatory bowel diseases undoubtedly requires further research, especially multicenter studies, which would increase credibility due to the size of the study group. Usually, the densitometry testing in IBD patients was performed with a delay, which could have influenced the collected data. This shows how early detection by timely performance DXA may be essential for correct diagnosis, therefore it requires more attention. It is crucial to identify the factors affecting bone metabolism and the mechanisms leading to reduced bone density, and to find ways to avoid the negative consequences on bone health, with the most optimal treatment outcomes for IBD.

## 5. Conclusions

Inflammatory bowel diseases in the pediatric population are becoming increasingly common, and often, the co-occurrence of lowered bone density is a crucial cause of concern. Studies have shown the importance of early diagnosis and appropriate monitoring of children with IBD, especially bone health and vitamin D levels. Moreover, biological factors such as CRP and calprotectin may also be useful in the process of examination and treatment. In addition, it is valid to identify and restrain the risk factors to protect bones from degradation and introduce a lifestyle that concentrates on strengthening the skeletal system. Given the high unpredictability of the development of the disease and its complications, further research is necessary to understand the relationships between IBD and bone metabolism, and effective preventive strategies may be developed. Enhancing our knowledge in this field can contribute to improving the quality of life and long-term health outcomes in children with IBD.

## Figures and Tables

**Table 1:** Clinical presentation of IBD ^1^ in children—Most frequently presented symptoms.

Presented Symptom	Classification of IBD, % of Patients		Significance
	Crohn’s Disease	Ulcerative Colitis	
Abdominal pain	85% (*n* = 23)	79% (*n* = 42)	*p* = 0.95
Diarrhea	59% (*n* = 16)	83% (*n* = 44)	***p* = 0.02**
Rectal bleeding	15% (*n* = 4)	91% (*n* = 48)	***p* < 0.001**
Weight loss	33% (*n* = 9)	32% (*n* = 17)	*p* = 0.83
Constipation	19% (*n* = 5)	13% (*n* = 7)	*p* = 0.49
Fever	30% (*n* = 8)	6% (*n* = 3)	*p* = 0.003

^1^ IBD—inflammatory bowel disease. Bolded *p*-values indicate statistically significant differences.

**Table 2:** Parameters analyzed among patients with IBD at the time of diagnosis—Comparison of UC and CD.

Parameter	Classification of IBD, Mean ± SD		Significance
	Crohn’s Disease (*n* = 27)	Ulcerative Colitis (*n* = 53)	
Age at diagnosis [years]	12.41 ± 3.35	12.10 ± 3.82	*p* = 0.80
CRP ^1^ [mg/L]	↑ 4.14 ± 8.03	↑ 1.05 ± 0.39	***p* < 0.001**
ESR ^2^ [mm/h]	↑ 42.36 ± 33.01	↑ 28.31 ± 23.97	***p* = 0.04**
Calcium [mmol/L]	2.24 ± 0.35	2.34 ± 0.17	*p* = 0.25
Inorganic Phosphate [mmol/L]	1.46 ± 0.33	1.43 ± 0.33	*p* = 0.39
Magnesium [mmol/L]	0.83 ± 0.05	0.82 ± 0.10	*p* = 0.97
Vitamin D [ng/mL]	↓ 24.5 ± 12.54	↓ 23.07 ± 9.20	*p* = 0.91
Parathormone [pg/mL]	25.13 ± 9.82	26.40 ± 12.22	*p* = 0.65
TSH [ulU/mL]	2.11 ± 0.97	2.07 ± 1.15	*p* = 0.36
FT4 [ng/mL]	1.30 ± 0.16	1.28 ± 0.21	*p* = 0.80
WBC ^3^ [thousands/μL]	9.54 ± 4.03	9.94 ± 3.51	*p* = 0.50
PLT ^4^ [thousands/μL]	435.58 ± 124.6	405.87 ± 124.15	*p* = 0.15
HGB ^5^ [g/dL]	↓ 11.69 ± 1.39	↓ 11.46 ± 1.79	*p* = 0.76
MCV ^6^ [fl]	76.05 ± 5.91	78.98 ± 6.45	***p* = 0.04**
Ferrum [ug/dL]	41.05 ± 26.87	50.02 ± 38.23	*p* = 0.61
ALP ^7^ [U/L]	142.07 ± 67.71	136.50 ± 78.57	*p* = 0.53
Z-Score TB ^8, a^ [Z-Points]	↓ −2.05 ± 1.62	↓ −1.27 ± 1.17	*p* = 0.55
Z-Score Spine ^a^ [Z-Points]	−0.78 ± 0.69	−0.63 ± 1.17	*p* = 0.78
Concentration of calprotectin in stool [mg/kg]	↑ 2258.9 ± 4343.0	↑ 3683.9 ± 7276.8	*p* = 0.14

^1^ CRP—C-reactive protein, ^2^ ESR—erythrocyte sedimentation rate, ^3^ WBC—white blood cells, ^4^ PLT—platelets, ^5^ hemoglobin, ^6^ mean cell volume, ^7^ alkaline phosphatase, ^8^ Total Body, ^a^—median time from diagnosis to densitometry, 208 days. ↑—above normal range, ↓—below normal range. Reference ranges: CRP < 0.5 mg/L, ESR < 15 mm/h, calcium 2.25–2.75 mmol/L, inorganic phosphate 0.9–1.6 mmol/L, magnesium 0.8–1.0 mmol/L, vitamin D 30–50 ng/mL, parathormone 4.8–40 pg/mL, PLT 150–450 thousands/μL, Z-Score TB and Z-Score Spine > −1.0 Z-Points, concentration of calprotectin in stool <50 mg/kg. Normal values of rest of parameters (TSH, FT4, WBC, HGB, MCV, Ferrum, ALP) depend on age and sex. Bolded *p*-values indicate statistically significant differences.

## Data Availability

The datasets generated and analyzed during the current study are not publicly available due to personal data protection, but are available from the corresponding author on reasonable request.
